# High-pressure water jet injury to the chest

**DOI:** 10.1016/j.tcr.2023.100847

**Published:** 2023-05-19

**Authors:** Koji Miura

**Affiliations:** Department of Emergency Medicine, Kurashiki Central Hospital, Japan

**Keywords:** Water jet injury, High-pressure water jet, Injection injury, Hydro blast injury

## Abstract

The high-pressure water jet cutting method, which uses a high-pressure water stream to cut hard materials, is widely used because it does not generate sparks or dust. However, once the high-pressure water jet is accidentally fired at a human body, a large amount of water containing abrasive materials flows into the body in a short time, causing severely contaminated injuries (Dailiana et al., 2008 [1]). Water jet injury (WJI) should be treated as a surgical emergency, but the severity is often underestimated, and treatment is delayed because the wound often shows only small holes [1]. Previous reports have shown that the majority of WJI occur in the extremities [1] (Rodríguez et al., 2019 [2]). On the other hand, abdominal and thoracic WJIs have been rarely reported, with only two cases of thoracic WJI [2]. Here, a case of thoracic WJI in which the patient was brought to our hospital the day after injury and treatment intervention was delayed is presented, and points to consider in the diagnosis and treatment strategy for WJI to the chest are discussed.

A 23-year-old man was injured by mishandling a gun-type high-pressure water jet while working at a concrete cutting site in the mountains. He was transported by a helicopter. When the flight doctor examined him, he was conscious and clear. An intercostal drainage was placed in the right thoracic cavity on suspicion of pneumothorax due to precordial trauma and complaints of dyspnea. He was transported to his previous doctor.

The left mandibular and right forearm lacerations were treated by the previous doctor. The chest injury was judged not to require additional intervention on the basis of computed tomography images, and intercostal drainage was continued. After being transferred to the ward, however, he complained of sensory and motor disturbance in the fingers of the right hand. He was transferred to our hospital the next day for orthopedic intervention.

Vital signs on admission to our hospital were as follows: respiratory rate 16 bpm; heart rate 96 bpm; blood pressure 128/65 mmHg; SPO_2_ 96 % (5 L/min via mask); body temperature 36.9 °C; and Glasgow Coma Scale score E4 M6 V5. The left mandibular and right forearm lacerations were reopened and cleaned. Surgery was scheduled for radial artery disruption and ulnar nerve transection. There were seven small holes with a diameter of 5 mm in the precordia (see Fig. 1), around which mild subcutaneous emphysema was palpable, but there was no bleeding or air leak, and no trauma to the back. A chest computed tomography image taken by the previous doctor showed a pulmonary contusion at the apex of the lungs (see Fig. 2) and extensive subcutaneous emphysema in the neck and chest (see Fig. 3).

**Fig. 1:**
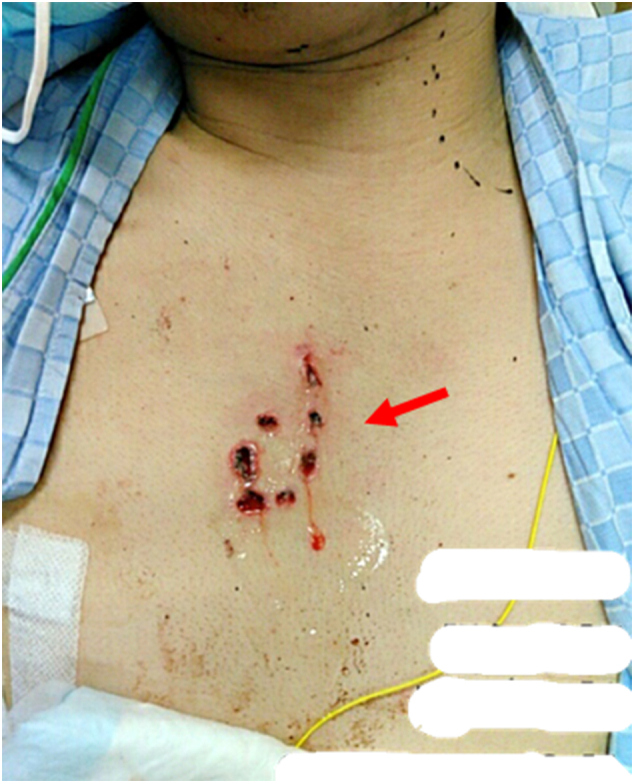
The seven small holes with a diameter of 5 mm in the precordia (red arrow). (For interpretation of the references to colour in this figure legend, the reader is referred to the web version of this article.)

**Fig. 2:**
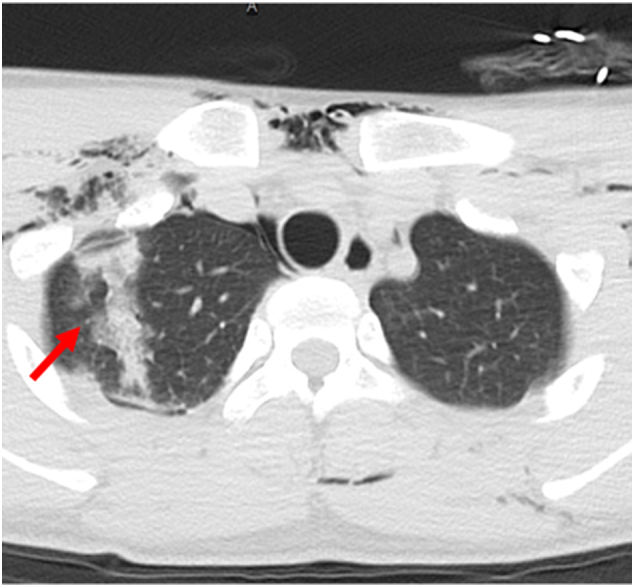
A chest computed tomography image taken by the previous doctor showing a pulmonary contusion at the apex of the lungs (red arrow). (For interpretation of the references to colour in this figure legend, the reader is referred to the web version of this article.)

**Fig. 3:**
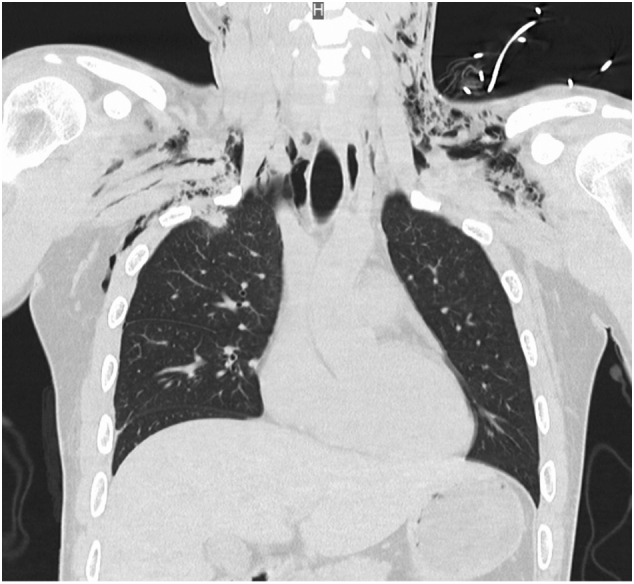
A chest computed tomography image taken by the previous doctor showing extensive subcutaneous emphysema in the neck and chest.

The precordial wound was small, and no further intervention was performed by the previous doctor; however, examination with forceps revealed that the inside of the water jet entry was severely contaminated with sandy foreign bodies. Surgical intervention for the chest wall was planned to assess the wound and perform debridement. After analgesia and sedation, one of the small holes in the precordia was incised toward the base of the wound, which revealed that the subcutaneous soft tissue had been peeled off by the high-pressure water flow and discolored as if burnt black. Removal of the sandy foreign bodies from the surrounding area revealed that pieces of clothing remained in the vicinity of the perforated pleura (see Fig. 4). The contaminated subcutaneous tissue was removed, and the wound was cleaned and closed. The subcutaneous injuries under the remaining six small holes, each of which was an isolated cavity, were all opened and debrided. A new intercostal drainage was placed through the lateral thoracic region to clean the thoracic cavity because of concerns about pyothorax due to contamination reaching the thoracic cavity. Subsequently, the intercostal drainage placed at the accident site was removed. In addition, the left mandibular laceration was reopened, debrided, and closed. Because the parotid duct was torn, compression was performed with a suppressor. The right forearm laceration was treated with reconstruction of the right ulnar nerve, suture of the extensor tendon of the hand, and thrombectomy of the radial artery. Ampicillin-sulbactam, which is effective against anaerobic bacteria, was selected as the perioperative antimicrobial agent.

**Fig. 4:**
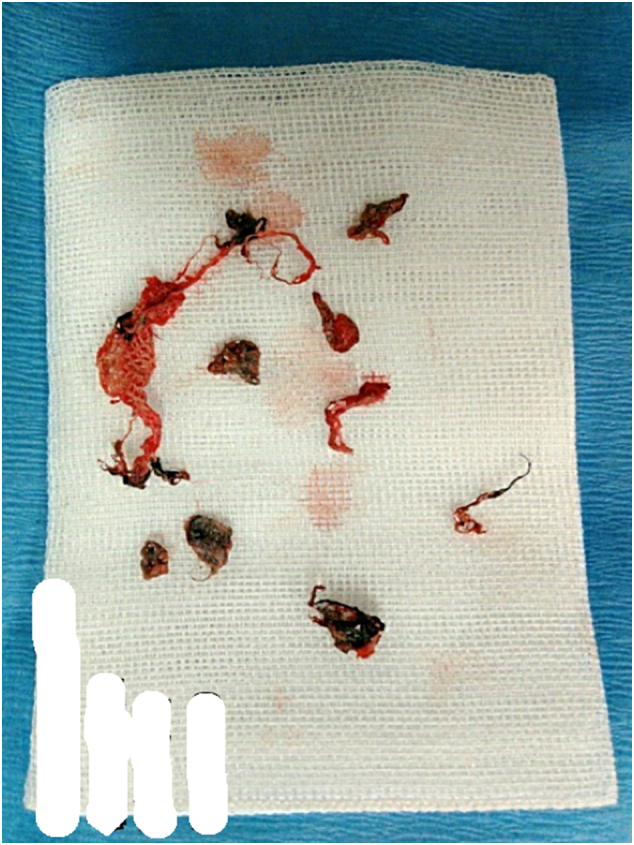
Pieces of clothing removed from the vicinity of the perforated pleura.

The postoperative course was uneventful, and a culture of the intercostal drainage effluent taken on the second postoperative day confirmed that no bacteria had developed. The patient did not develop pyothorax thereafter. Sensory and motor disturbance in the fingers of the right hand gradually improved, and he was discharged home on the 30th postoperative day. At the time of writing, 12 months have passed since the surgery, and he has returned to work without any complications.

## Discussion

WJI is a surgical emergency in which tissue contusion and severe contamination coexist [[3], [4], [5], [6]]. In real-world clinical settings, however, thoracic WJI is rare among WJIs overall [1,2] and is not well recognized as a severe trauma, which may be one of the reasons for the delay in therapeutic intervention. The wound mechanism of WJI is similar to that of gunshot injury, but is characterized by a wide, blunt dissection of the subcutaneous tissue due to a large amount of water injected into the tissue in a short period of time. As with gunshot injury, WJI is contaminated and should be kept open. It is also essential to assess the damage with aggressive surgical intervention, even if the wound is small, because entries without exits tend to be more severe [2]. Extremity trauma can be easily opened but not chest trauma, and the latter requires careful debridement and autologous tissue closure or postoperative negative pressure closure therapy [7]. Surgical intervention should be therefore performed promptly to control infection, but care must be taken not to underestimate the extent of the injury and not to complicate wound closure.

In the treatment of thoracic WJI, computed tomography images are useful for determining whether the thoracic cavity is involved or not. Even in the absence of pneumothorax or pleural effusion on computed tomography imaging, the presence of a localized pulmonary contusion can be diagnostic of fluid flow across the pleura. A previous case report showed focal linear lung injury [8], which we consider to be one of the findings to determine the presence or absence of pleural perforation in a thoracic WJI. If the pleura is perforated, the thoracic cavity should be cleaned because contaminated water or pieces of clothing may remain there [9,10]. Although thoracic lavage can be performed through an implanted drain, thoracoscopic surgery under general anesthesia is more reliable.

In this case, treatment was initiated from the chest wall tissue. Because the foreign bodies in the operative field, including pieces of clothing, were finely torn, it was unlikely that any foreign bodies that exceeded the drain's inner diameter entered the thoracic cavity. In addition, because the patient was a young man with no underlying disease and low risk of infection, two 28-Fr thoracic drains were used for cleaning, and thoracoscopic surgery was not performed. Not all cases require thoracoscopic surgery, but thoracoscopic lavage should be considered for highly infectious or severely contaminated patients [4]. Further case accumulation is needed in this regard.

## Conclusion

WJI is a rare severe trauma that is often underestimated and delayed in treatment. Although the number of cases of thoracic WJI is small, it is a surgical emergency that requires a treatment strategy based on infection control and wound closure, and aggressive surgical intervention should not be hesitated.

## Declaration of competing interest

The authors declare that they have no known competing financial interests or personal relationships that could have appeared to influence the work reported in this paper.
